# Shared genomic architecture between COVID-19 severity and numerous clinical and physiologic parameters revealed by LD score regression analysis

**DOI:** 10.1038/s41598-022-05832-5

**Published:** 2022-02-03

**Authors:** Jinyoung Byun, Younghun Han, Kyle M. Walsh, Amy S. Park, Melissa L. Bondy, Christopher I. Amos

**Affiliations:** 1 Institute for Clinical and Translational Research, Baylor College of Medicine, Houston, TX USA; 2 Section of Epidemiology and Population Sciences, Department of Medicine, Baylor College of Medicine, Houston, TX USA; 3 Duke Cancer Institute, Duke University Medical Center, Durham, NC USA; 4 Department of Epidemiology and Population Health, School of Medicine, Stanford University, Stanford, CA USA; 5 Dan L Duncan Comprehensive Cancer Center, Baylor College of Medicine, Houston, TX USA

**Keywords:** Genome-wide association studies, Genetic predisposition to disease

## Abstract

The COVID-19 pandemic has produced broad clinical manifestations, from asymptomatic infection to hospitalization and death. Despite progress from genomic and clinical epidemiology research, risk factors for developing severe COVID-19 are incompletely understood and identification of modifiable risk factors is desperately needed. We conducted linkage disequilibrium score regression (LDSR) analysis to estimate cross-trait genetic correlation between COVID-19 severity and various polygenic phenotypes. To attenuate the genetic contribution of smoking and BMI, we further conducted sensitivity analyses by pruning genomic regions associated with smoking/BMI and repeating LDSR analyses. We identified robust positive associations between the genetic architecture of severe COVID-19 and both BMI and smoking. We observed strong positive genetic correlation (rg) with diabetes (rg = 0.25) and shortness of breath walking on level ground (rg = 0.28) and novel protective associations with vitamin E (rg = − 0.53), calcium (rg = − 0.33), retinol (rg = − 0.59), Apolipoprotein A (rg = − 0.13), and HDL (rg = − 0.17), but no association with vitamin D (rg = − 0.02). Removing genomic regions associated with smoking and BMI generally attenuated the associations, but the associations with nutrient biomarkers persisted. This study provides a comprehensive assessment of the shared genetic architecture of COVID-19 severity and numerous clinical/physiologic parameters. Associations with blood and plasma-derived traits identified biomarkers for Mendelian randomization studies to explore causality and nominates therapeutic targets for clinical evaluation.

## Introduction

Since the outbreak of severe acute respiratory syndrome coronavirus 2 (SARS-CoV-2) in December 2019, the global pandemic of coronavirus disease 2019 (COVID-19) has resulted in more than 205 million confirmed cases and 4.3 million deaths worldwide as of August 13, 2021^1^. Genome-wide association studies (GWAS) have revealed the genetic underpinnings of complex human traits, helping to elucidate the heritability of susceptibility to both chronic diseases and infectious diseases—including COVID-19^2^. Such complex diseases typically have multifactorial etiologies, with contributions from germline genetic variation and environmental exposures^3^, and they frequently present with related, comorbid medical conditions^4^.

SARS-CoV-2 is a highly contagious respiratory virus that also impacts additional organ systems. Several comorbid risk factors are known to be associated with developing severe COVID-19 illness^5^, including hypertension, cardiovascular disease, chronic obstructive pulmonary disease (COPD), high body mass index (BMI), and type 2 diabetes^6^. Diabetes, associated with immunosuppression and vascular and renal complications, has emerged as a critical comorbidity among severely ill COVID-19 patients^7^.

Leveraging GWAS data, we conducted cross-trait genetic correlation analyses to examine the sum effect of pleiotropy across all causal loci to determine if we could reveal shared genetic correlations between multiple polygenic traits. In addition, we determined the directionality of these associations, and whether the genetic architecture of two traits are correlated or anti-correlated^8^. Examining the shared genetic architecture underlying co-occurrence of disease can help elucidate their common genetic etiology^4^, but there has been no comprehensive evaluation of the shared genetic architecture between COVID-19 disease severity and additional diseases or traits. Previous analyses of lung cancer risk reveal shared genetic architecture with emphysema, a common co-morbid condition, as well as with cigarette consumption, the leading cause of lung cancer^4^. In the setting of COVID-19 disease severity, such an evaluation has similar potential to identify co-morbid conditions and to uncover traits that are causally involved in worsening clinical course.

To identify polygenic traits sharing underlying genetic etiology with COVID-19 disease severity, we utilized summary statistics from prior GWAS and conducted cross-trait linkage disequilibrium (LD) score regression (LDSR) analyses^9,10^. Results provide fundamental knowledge on traits and conditions that share genetic underpinnings with COVID-19 disease severity, reveal potential risk factors for developing severe COVID-19 disease subsequent to SARS-CoV-2 infection, and implicate several modifiable factors that merit further study and may ultimately help improve patient outcomes.

## Methods

### GWAS summary statistics for COVID-19 critical illness and hospitalization

We downloaded the GWAS summary statistics (COVID19-hg GWAS meta-analyses round 5, released on January 18, 2021; https://www.covid19hg.org/results/r5/) from the COVID-19 Host Genetics Initiative (COVID-19 HGI)^11–13^, comprising (1) A2 (critical illness)^13^: 4,606 very severe, respiratory-confirmed COVID-19 patients versus 702,801 population-based controls (A2_ALL_eur_leave_23andme) and (2) B2 (hospitalization)^13^: 9,373 hospitalized COVID-19 patients versus 1,197,256 population-based controls (B2_ALL_eur_leave_23andme) (Table 1 and Supplementary Tables 1 and 2). While the summary statistics of COVID-19-hg GWAS meta-analyses across multiple populations have been deposited at the COVID-19 HGI^11,13^, we restricted analyses to European-ancestry subjects (to align with the ancestral background of participants in GWAS of traits used in our downstream LDSR analyses) and did not include the 23andMe cohort (due to the data-use constraint, which makes only the top 10,000 SNPs publicly available)^14^. All methods were performed in accordance with the relevant guidelines and regulations.

**Table 1:** Study description.

Strata	Trait	Sample size	SNPs
A2 very severe, respiratory-confirmed COVID-19 patients versus population-based controls	COVID-19 A2	707,407	1,140,193
B2 hospitalized COVID-19 patients versus population-based controls	COVID-19 B2	1,206,629	1,141,302
**Exclusion of smoking-associated genomic regions**			
A2 very severe, respiratory-confirmed COVID-19 patients versus population-based controls	COVID-19 A2⟂Smoke	707,407	1,001,866
B2 hospitalized COVID-19 patients versus population-based controls	COVID-19 B2⟂Smoke	1,206,629	1,003,099
**Exclusion of BMI-associated genomic regions**			
A2 very severe, respiratory-confirmed COVID-19 patients versus population-based controls	COVID-19 A2⟂BMI	707,407	856,012
B2 hospitalized COVID-19 patients versus population-based controls	COVID-19 B2⟂BMI	1,206,629	856,864

### GWAS summary statistics for additional traits

To estimate cross-trait genetic correlation patterns between COVID-19 disease severity and multiple polygenic traits, we harmonized publicly available GWAS summary-level data from the UK Biobank (UKBB), a prospective population-based cohort study consisting of ~ 500,000 individuals, aged 40–69 years, who were recruited in the United Kingdom between 2006 and 2010^15,16^. All methods were carried out in accordance with relevant guidelines and regulations.

GWAS summary-level data used for the LDSR analyses of UKBB traits are from publicly-posted results generated by the Neale lab (http://www.nealelab.is/uk-biobank/). These association analyses are adjusted with the first 20 principal components, which adjust for sources of population level variability in genetic allele frequencies. The GWAS summary-level data of UKBB used in our study are restricted to “British ancestry” using the first 6 principal components to determine “British ancestry” and further filtered by self-reported ethnicity with “white-British”, “Irish”, or “White”. The sample sizes and more details for the tested traits are shown in Supplementary Table 2.

### Estimating SNP-heritability and cross-trait genetic correlation of COVID-19

LD score regression analysis with 1000 Genomes Project European (EUR) samples as a reference for pattern of genome-wide LD quantifies the co-heritability of diverse traits^4,9,10,17^ using GWAS summary statistics for common genetic variants (i.e., SNPs). In brief, LDSR method regresses χ^2^ statistics from GWAS on LD scores, allowing the estimation of genetic correlation without bias due to population stratification or cryptic relatedness^4,9,10,18,19^. By regressing SNP-level associations for two traits, (i.e., the product of Z scores, Z_COVID19_A2_ × Z_UKBB_BMI_) and weighting each SNP by its LD Score (an estimate of the amount of total genetic variation tagged by each variant), one can estimate the magnitude and direction of shared genomic architecture between these traits. To control the multiple testing burden, we restricted analyses to the tested UKBB traits showing heritability ≥ 1% and for which prior studies have suggested correlations between COVID-19 and risk for severe outcomes, or traits that were correlated with traits that have been associated with severe outcomes. We conservatively set the test-wise level of significance after Bonferroni correction to be 0.05/(6 × 64), adjusting for analysis of COVID-19 severity (A2 and B2) with 64 UKBB traits, with and without removal of BMI and Smoking SNPs. We first implemented the command option of LD Score (https://github.com/bulik/ldsc; ldsc v1.0.1) with “munge_sumstats.py” to generate the “.sumstats” format from the GWAS summary statistics after ~ 1.14 M HapMap3 SNPs with MAF > 1% were selected for the analysis as recommended. Multi-allelic SNPs and the major histocompatibility complex (MHC) region (Chr6:25–34 Mb) were excluded from summary statistics because of the complex and unusual LD pattern and genetic architecture of the MHC region^4^. We then applied “ldsc.py -rg covid19.A2.sumstats.gz, trait1.sumstats.gz-ref-ld-chr eur_w_ld_chr/-w-ld-chr eur_w_ld_chr/-out covid19.A2_triat1”.

### Exclusion of genomic regions related to smoking behavior and BMI

Although a clearer picture is emerging, the contribution of cigarette smoking to COVID-19 disease severity remains incompletely understood, with most studies suggesting increased disease severity among former smokers versus never-smokers, but some studies observing a protective effect for current smoking^20^ and others showing an increased risk for more severe symptoms in smokers^21^. Since smoking behaviors are heritable traits that correlate with many other complex diseases, we performed sensitivity analyses by excluding chromosomal regions (± 500 kb) around 473 SNPs previously associated with various smoking behaviors (⟂Smoke) to attenuate the genetic contribution of smoking-related variants^4^. The removed genomic regions related to cigarettes per day, smoking initiation, smoking cessation, initiation age of regular smoking, and nicotine dependence (Supplementary Tables 3 and 4).

Although obesity increases risk of systemic inflammation, pulmonary clots, stroke, and myocardial infarction, it remains unclear whether reported associations between BMI and COVID-19 disease severity are confounded by socioeconomic status or concurrent health issues. We performed sensitivity analyses by excluding genomic regions (± 500 kb) around 941 SNPs previously associated with BMI (⟂BMI) to attenuate the genetic contribution of BMI-related variants (Supplementary Tables 4 and 5).

## Results

**Figure 1:**
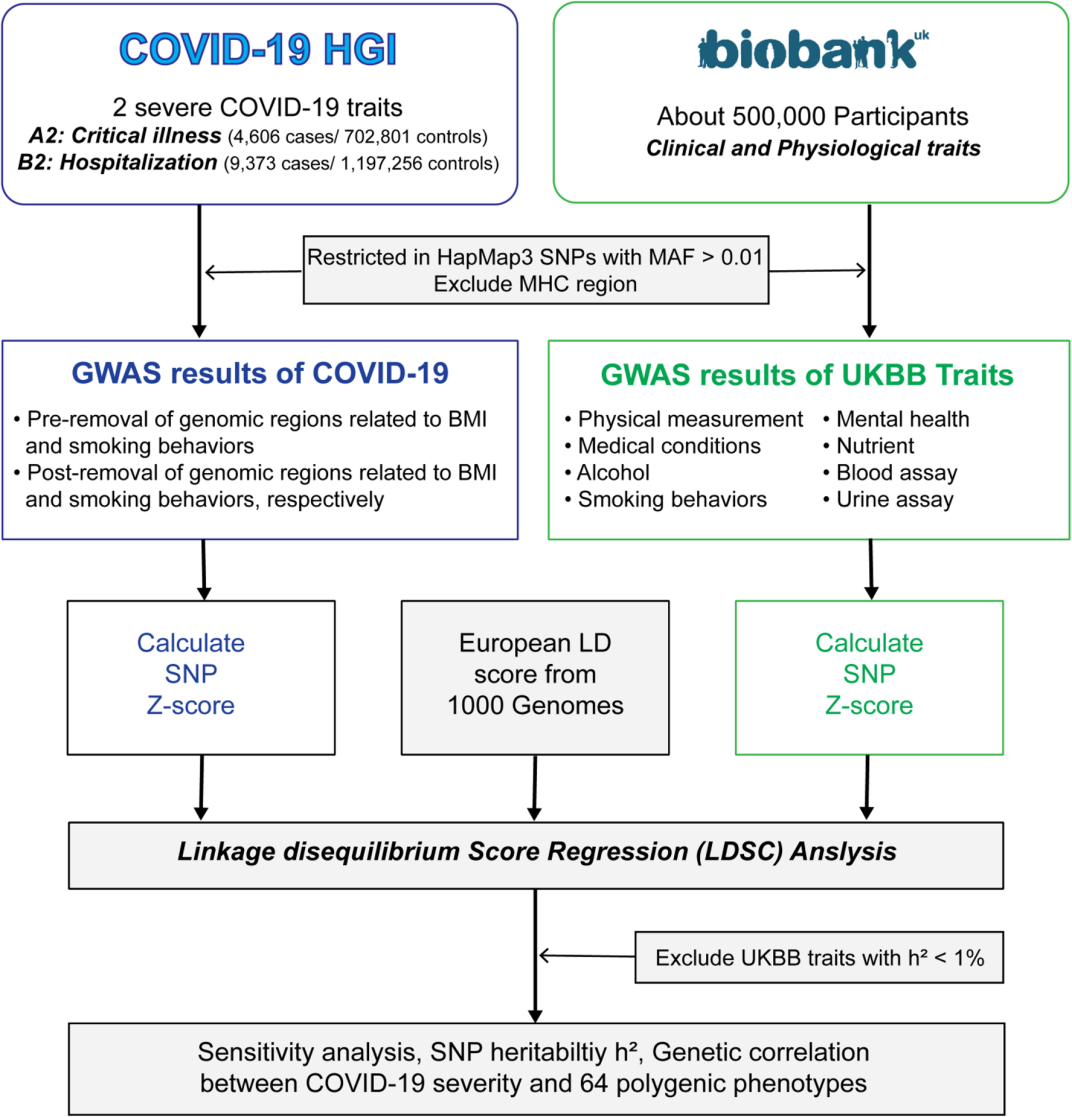
Flow chart of the analytical workflow in the study.

**Figure 2:**
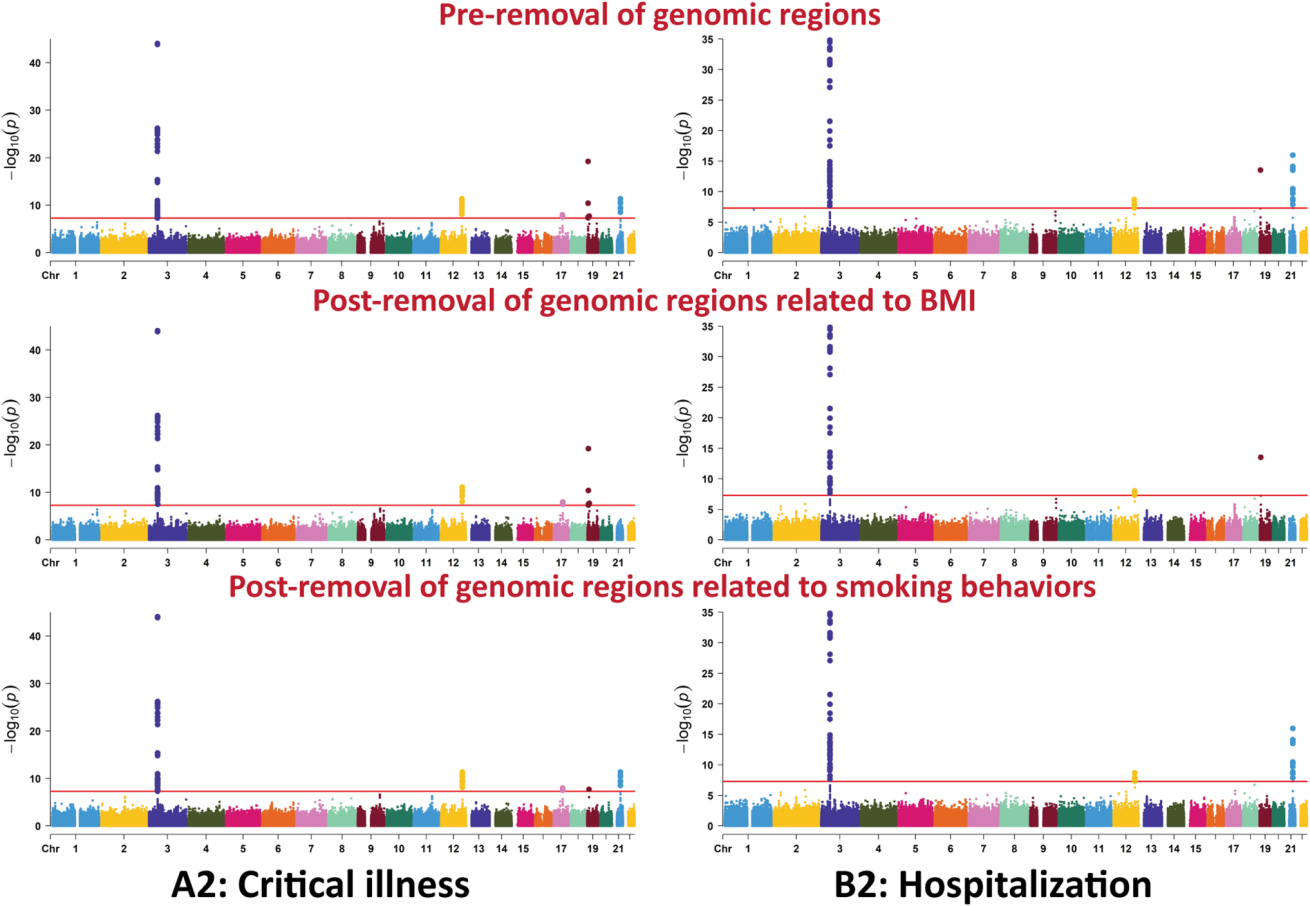
Manhattan plots of the COVID-19 GWAS meta-analysis for pre- and post-removal of genomic regions associated with smoking behaviors and BMI in European descnt population. A2: very severe respiratory confirmed COVID-19 cases versus population: 4606 cases and 702,801 controls, B2: hospitalized COVID-19 cases versus population: 9373 cases and 1,197,256 controls.

We implemented cross-trait LDSR analysis to examine shared genetic contributions to COVID-19 disease severity and multiple clinical and epidemiologic traits using pairwise genetic correlations (rg) and the observed-scale heritability (h^2^, representing the proportion of phenotypic variance explained by all common SNPs). The flow chart presented in Fig. 1 summarizes the steps from data preparation to LDSR analysis for COVID-19 severity versus 64 polygenic traits we studied. A prior GWAS analysis^13^ of very severe, respiratory-confirmed COVID-19 (phenotype A2: critical illness; 4606 cases, 702,801 controls in only European descent) identified loci on chromosomes 3, 12, 17, 19, and 21 that reached genome-wide statistical significance (*P* < 5.0 × 10^−8^ shown in the red horizontal line), with a genomic inflation factor of 1.047, and an estimated h^2^ of 0.35%. Sensitivity analysis excluding chromosomal regions known to be associated with smoking reduced the genomic inflation factor to 1.041 and h^2^ to 0.34%. Sensitivity analysis excluding chromosomal regions known to be associated with BMI increased the genomic inflation factor to 1.050 and h^2^ to 0.35% (Fig. 2, Supplementary Table 6).

A prior GWAS analysis of hospitalized COVID-19 (phenotype B2: hospitalization; 9373 cases, 1,197,256 controls in only European-descent) identified loci on chromosomes 3, 12, 19, and 21 at genome-wide statistical significance, with a genomic inflation factor of 1.041, and an estimated h^2^ of 0.19% (Fig. 2). Sensitivity analysis excluding chromosomal regions known to be associated with smoking reduced the genomic inflation factor to 1.038 and h^2^ to 0.19%. Sensitivity analysis excluding chromosomal regions known to be associated with BMI reduced the genomic inflation factor to 1.035 and h^2^ to 0.17% (Fig. 2, Supplementary Table 6).

**Figure 3:**
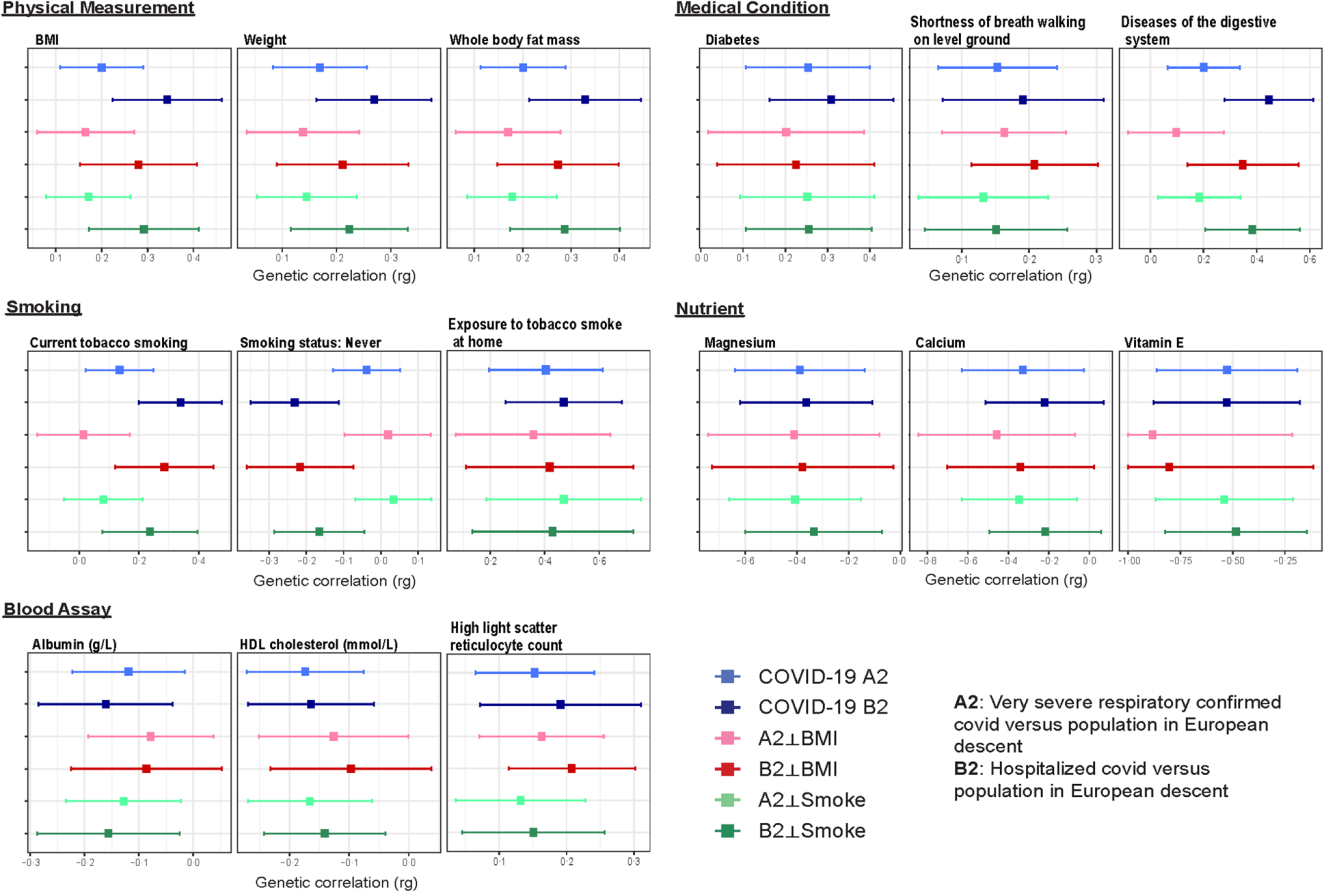
The pairwise genetic correlation of COVID-19 disease severity and selected traits.

Using these GWAS results, we next performed LDSR analyses with two phenotypes for COVID-19 severity (COVID-19 A2, and COVID-19 B2) considering four phenotypes for exclusions of genomic regions related to BMI and Smoking (COVID19_A2⟂BMI, COVID19_A2⟂Smoke, COVID19_B2⟂BMI, and COVID19_B2⟂Smoke) and 64 UKBB polygenic traits that had SNP array-based heritability (h^2^) ≥ 1% (to maximize study power and to provide reliable inferences). Twenty-three diverse traits showed moderate to strong co-heritability with COVID-19 disease severity (Table 2, Supplementary Table 7), including several at Bonferroni-corrected significance level (*P* < 1.30 × 10^−4^). Very severe, respiratory-confirmed COVID-19 illness (A2) and COVID-19 hospitalization (B2) showed strong genomic correlation with traits related to adiposity, diabetes, digestive diseases, smoking behaviors, hematologic traits, and selected nutrient levels (Fig. 3, Supplementary Table 7).

**Table 2:** Cross-trait genetic correlations of COVID-19 on inclusion/exclusion of genomic regions associated with BMI and smoking.

Traits	COVID-19 critical illness (A2)						COVID-19 hospitalization (B2)					
	A2		A2⟂BMI		A2⟂Smoke		B2		B2⟂BMI		B2⟂Smoke	
	rg	*P*	rg	*P*	rg	*P*	rg	*P*	rg	*P*	rg	*P*
**Physical measurement**												
BMI	0.200	**1.51 × 10**^−**5**^	0.165	2.37 × 10^−3^	0.171	3.07 × 10^−4^	0.343	**1.99 × 10**^−**8**^	0.280	**1.82 × 10**^−**5**^	0.292	**1.42 × 10**^−**6**^
Weight	0.169	**1.24 × 10**^−**4**^	0.138	9.60 × 10^−3^	0.145	2.24 × 10^−3^	0.269	**7.23 × 10**^−**7**^	0.211	6.86 × 10^−4^	0.223	**4.23 × 10**^−**5**^
Whole body fat mass	0.201	**7.81 × 10**^−**6**^	0.170	2.00 × 10^−3^	0.178	1.49 × 10^−4^	0.329	**2.24 × 10**^−**8**^	0.273	**2.32 × 10**^−**5**^	0.287	**8.51 × 10**^−**7**^
**Medical condition**												
Shortness of breath walking on level ground	0.283	2.87 × 10^−3^	0.198	0.107	0.272	8.33 × 10^−3^	0.433	**4.56 × 10**^−**5**^	0.372	5.94 × 10^−3^	0.395	4.01 × 10^−4^
Diabetes diagnosed by doctor	0.254	7.10 × 10^−4^	0.202	0.032	0.252	1.95 × 10^−3^	0.309	**3.39 × 10**^−**5**^	0.225	0.018	0.256	8.00 × 10^−4^
Diagnoses—main ICD10: K57 Diverticular disease of intestine	0.297	4.31 × 10^−4^	0.228	0.019	0.315	6.64 × 10^−4^	0.380	**1.66 × 10**^−**5**^	0.305	2.84 × 10^−3^	0.355	2.01 × 10^−4^
Diseases of the digestive system	0.201	3.48 × 10^−3^	0.097	0.292	0.183	0.020	0.446	**1.86 × 10**^−**7**^	0.348	1.14 × 10^−3^	0.384	**2.30 × 10**^−**5**^
Diseases of the musculoskeletal system and connective tissue	0.243	4.84 × 10^−4^	0.121	0.170	0.223	5.96 × 10^−3^	0.338	**3.54 × 10**^−**6**^	0.174	0.038	0.272	4.62 × 10^−4^
**Smoking**												
Current tobacco smoking	0.135	0.021	0.014	0.858	0.081	0.223	0.339	**2.01 × 10**^−**6**^	0.285	6.57 × 10^−4^	0.237	3.33 × 10^−3^
Exposure to tobacco smoke at home	0.404	1.67 × 10^−4^	0.358	0.013	0.470	1.16 × 10^−3^	0.470	**1.73 × 10**^−**5**^	0.418	7.79 × 10^−3^	0.429	4.62 × 10^−3^
**Nutrient**												
Magnesium	− 0.389	2.28 × 10^−3^	− 0.411	0.015	− 0.407	1.81 × 10^−3^	− 0.364	5.17 × 10^−3^	− 0.378	0.035	− 0.335	0.013
Calcium	− 0.329	0.033	− 0.457	0.020	− 0.347	0.017	− 0.222	0.137	− 0.340	0.067	− 0.219	0.121
Retinol	− 0.591	0.041	− 0.888	0.027	− 0.484	0.081	− 0.588	0.029	− 1.00	0.018	− 0.473	0.076
Vitamin E	− 0.527	2.16 × 10^−3^	− 0.883	0.010	− 0.540	1.26 × 10^−3^	− 0.528	3.10 × 10^−3^	− 0.804	0.022	− 0.484	5.08 × 10^−3^
**Blood Assay**												
Albumin (g/L)	− 0.119	0.026	− 0.071	0.229	− 0.128	0.017	− 0.161	0.011	− 0.086	0.225	− 0.156	0.020
Apoliprotein A (g/L)	− 0.134	6.17 × 10^−3^	− 0.093	0.138	− 0.126	0.016	− 0.118	0.024	− 0.061	0.369	− 0.101	0.047
C-reactive protein (mg/L)	0.187	1.88 × 10^−4^	0.139	0.015	0.218	1.50 × 10^−4^	0.278	3.04 × 10^−4^	0.237	2.79 × 10^−4^	0.294	**2.98 × 10**^−**5**^
HDL cholesterol	− 0.174	5.20 × 10^−4^	− 0.126	0.048	− 0.166	1.60 × 10^−3^	− 0.164	2.25 × 10^−3^	− 0.097	0.161	− 0.141	6.93 × 10^−3^
High light scatter reticulocyte count	0.153	7.13 × 10^−4^	0.163	5.33 × 10^−4^	0.132	6.74 × 10^−3^	0.208	**1.23 × 10**^−**5**^	0.191	1.83 × 10^−3^	0.151	4.73 × 10^−3^
High light scatter reticulocyte percentage	0.155	4.13 × 10^−4^	0.168	1.78 × 10^−4^	0.131	5.68 × 10^−3^	0.209	**7.66 × 10**^−**6**^	0.198	8.04 × 10^−4^	0.150	5.12 × 10^−3^
IGF-1 (nmol/L)	− 0.148	4.99 × 10^−3^	− 0.155	0.017	− 0.153	5.64 × 10^−3^	− 0.090	0.077	− 0.116	0.064	− 0.083	0.118
Immature reticulocyte fraction	0.173	1.58 × 10^−3^	0.156	0.010	0.155	6.05 × 10^−3^	0.264	**1.05 × 10**^−**5**^	0.219	3.92 × 10^−3^	0.199	3.59 × 10^−3^
Platelet distribution width	− 0.108	0.017	− 0.130	0.011	− 0.109	0.020	− 0.038	0.440	− 0.060	0.256	− 0.038	0.432

*P*-values in bold indicates *P* ≤ 1.30 × 10^−4^. COVID19_A2, very severe respiratory confirmed covid versus population including whole genomic regions; A2⟂BMI, very severe respiratory confirmed covid versus population with exclusion of genomic regions related to BMI; A2⟂Smoke, very severe respiratory confirmed covid versus population with exclusion of genomic regions related to smoking behaviors; COVID19_B2, hospitalized covid versus population including whole genomic regions; B2⟂BMI, hospitalized covid versus population with exclusion of genomic regions related to BMI; B2⟂Smoke, hospitalized covid versus population with exclusion of genomic regions related to smoking behaviors.

Among physical traits, the genetic architecture of COVID-19 disease severity was positively correlated with BMI (rg_COVID19_A2_ = 0.20, P_COVID19_A2_ = 1.51 × 10^−5^; rg_COVID19_B2_ = 0.34, P_COVID19_B2_ = 1.99 × 10^−8^), weight (rg_COVID19_A2_ = 0.17, P_COVID19_A2_ = 1.24 × 10^−4^; rg_COVID19_B2_ = 0.27, P_COVID19_B2_ = 7.23 × 10^−7^), and whole body fat mass (rg_COVID19_A2_ = 0.20, P_COVID19_A2_ = 7.81 × 10^−6^; rg_COVID19_B2_ = 0.33, P_COVID19_B2_ = 2.24 × 10^−8^). After excluding genomic regions previously associated with BMI, both BMI and whole body fat mass continued to show strongly significant positive correlation with COVID-19 disease severity (rg_COVID19_A2⟂BMI_ = 0.17 and P_COVID19_A2⟂BMI_ = 2.37 × 10^−3^; rg_COVID19_B2⟂BMI_ = 0.28 and P_COVID19_B2⟂BMI_ = 1.82 × 10^−5^).

Among medical conditions, the genetic architecture of COVID-19 disease severity was positively correlated with shortness of breath walking on level ground (rg_COVID19_A2_ = 0.28, P_COVID19_A2_ = 2.87 × 10^−3^; rg_COVID19_B2_ = 0.43, P_COVID19_B2_ = 4.56 × 10^−5^), diabetes (rg_COVID19_A2_ = 0.54, P_COVID19_A2_ = 7.10 × 10^−4^; rg_COVID19_B2_ = 0.31, P_COVID19_B2_ = 3.39 × 10^−5^), diverticulosis (rg_COVID19_A2_ = 0.30, P_COVID19_A2_ = 4.31 × 10^−4^; rg_COVID19_B2_ = 0.38, P_COVID19_B2_ = 1.66 × 10^−5^), diseases of the digestive system (rg_COVID19_A2_ = 0.20, P_COVID19_A2_ = 3.48 × 10^−3^; rg_COVID19_B2_ = 0.45, P_COVID19_B2_ = 1.86 × 10^−7^), and diseases of the musculoskeletal system and connective tissue (rg_COVID19_A2_ = 0.24, P_COVID19_A2_ = 4.84 × 10^−4^; rg_COVID19_B2_ = 0.34, P_COVID19_B2_ = 3.54 × 10^−6^). Excluding genomic regions associated with smoking behaviors or BMI generally attenuated these correlations, although most remained nominally associated at P < 0.05 and the association between COVID-19 hospitalization and diseases of the digestive system remained significant at Bonferroni-corrected levels after exclusion of smoking-associated loci (rg_COVID19_B2⟂Smoke_ = 0.38, P_COVID19_B2⟂Smoke_ = 2.30 × 10^−5^).

Among smoking behaviors, current tobacco smoking (rg_COVID19_B2_ = 0.34, P_COVID19_B2_ = 2.01 × 10^−6^) and exposure to tobacco smoke at home (rg_COVID19_B2_ = 0.47, P_COVID19_B2_ = 1.73 × 10^−5^) presented strongly significant positive genetic correlation with COVID-19 hospitalization, which were only modestly attenuated when removing known smoking-associated loci from analysis. Current tobacco smoking was more modestly associated with severe, respiratory-confirmed COVID-19 illness COVID (rg_COVID19_A2_ = 0.13, P_COVID19_B2_ = 0.021) and this association became non-significant after removing smoking-associated loci from analysis (Table 2), supporting a link between known smoking risk loci and risk for severe COVID-19 outcomes.

Examining hematologic traits, both high light scatter reticulocyte percentage and count were significantly positively correlated with COVID-19 hospitalization, as was immature reticulocyte fraction. These traits were also positively correlated with severe COVID-19 illness, but not at Bonferroni-corrected levels of statistical significance. C reactive protein levels were also positively correlated with COVID-19 disease severity. Interestingly, serum (not urinary) albumin was negatively correlated with COVID-19 disease severity at nominal statistical significance (rg_COVID19_A2_ = − 0.12, P_COVID19_A2_ = 0.026; rg_COVID19_B2_ = − 0.16, P_COVID19_B2_ = 0.011), as were HDL, apolipoprotein A levels, and levels of serum IGF-1 (Table 2).

We also examined the pairwise genetic relationship between COVID-19 disease severity and nutrient-related traits in UKB. Although we were not able to observe any significant associations between COVID-19 critical illness and hospitalization and nutrient-related traits at Bonferroni-corrected levels, we identified suggestive negative correlations with magnesium (rg_COVID19_A2_ = − 0.39, P_COVID19_A2_ = 2.28 × 10^−3^; rg_COVID19_B2_ = − 0.36, P_COVID19_B2_ = 5.17 × 10^−3^), retinol (rg_COVID19_A2_ = − 0.59, P_COVID19_A2_ = 0.041; rg_COVID19_B2_ = − 0.59, P_COVID19_B2_ = 0.029), and vitamin E (rg_COVID19_A2_ = − 0.53, P_COVID19_A2_ = 2.16 × 10^−3^; rg_COVID19_B2_ = − 0.53, P_COVID19_B2_ = 3.10 × 10^−3^) (Table 2 and Supplementary Table 7). Vitamin D levels were not associated with risk for severe COVID-19 (rg_COVID19_A2_ = − 0.023, P_COVID19_A2_ = 0.67; rg_COVID19_B2_ = − 0.043, P_COVID19_B2_ = 0.44).

## Discussion

We investigated the genetic correlations between COVID-19 disease severity (A2:critical illness and B2:hospitalization) with a variety of clinical and physiologic traits using summary-level GWAS data from extremely large patient cohorts, observing shared genomic architecture with a number of illnesses and biomarkers of somatic well-being. We identify a suite of medical conditions and physiological traits that appear to share the genetic architecture with that of COVID-19 severity. Many of these traits overlap those previously identified in the large databases of COVID-19 patient outcomes, including traits related to adiposity, kidney function, and pulmonary insufficiency. We also identified additional traits that have received comparatively little attention, such as blood and serum levels of several vitamins and nutrients. Although our datasets are quite large (COVID-19 severity GWAS n = 707,407 and 1,206,629 for critical illness (A2) and hospitalization (B2), respectively; UKBB GWAS n = 361,194), larger datasets would likely identify many of these same associations and could potentially bring some of the nominally associated associations to a corrected level of statistical significance.

Using an orthogonal genomics-driven approach that complements previous COVID-19 clinical epidemiology research, we confirm a link between the development of severe COVID-19 illness and both elevated BMI and diabetes. We also clarify associations with current smoking status, observing that it was positively correlated with COVID-19 disease severity, and note new associations with diverticulosis and reticulocyte traits. Additionally, we observe a suggestive association between increased disease severity and reduced levels of IGF-1—a marker of nutritional status—and additional suggestive protective associations with magnesium, retinol, and vitamin E levels.

COVID-19 is primarily a respiratory illness. We observed that higher forced vital capacity (FVC) was negatively (protectively) associated with COVID-19 disease severity and observed a strongly positive correlation between the genetic architecture of ‘shortness of breath while walking on level ground’ and development of severe COVID-19 illness. Chest pain and discomfort have previously been associated with COVID-19 hospitalization and the U.S. Centers for Disease Control and Prevention (CDC) announced that individuals with chronic lung diseases including emphysema, chronic bronchitis, COPD, and interstitial lung disease are at high risk for becoming critically ill from SARS-CoV-2^1^. Our study demonstrates a positive correlation between the genetic architecture of these risk factors and COVID-19 disease severity through LDSR analyses. In this study, a differential diagnosis of COPD was strongly positively correlated with COVID-19 hospitalization, regardless of the exclusion of genomic regions related to BMI and smoking behaviors. Since chronic inflammation is an important feature in developing both emphysema and bronchitis, these finding suggest a potential shared genetic contribution between COPD and COVID-19 hospitalization separate from the contributions of known BMI and smoking-related variants. Variants located in immune-related genes and contributing to increased pulmonary inflammation could be evaluated in future work.

Traits related to smoking behaviors were generally associated with increased COVID-19 disease severity in our analyses, including current smoking, exposure to tobacco smoke either at home or outside home, in utero tobacco smoke exposure, and cumulative pack-years. Conversely, never-smoker status showed negative genomic correlation with COVID-19 disease severity. Although UKBB does not delineate former smokers in ascribing smoking status, our analyses indicate that the genetic determinants of current smoking are associated with increased COVID-19 disease severity and do not support the clinical observations that current smoking may protect against severe COVID-19 illness.

Given the lack of a COVID-19 vaccine during the first year of the pandemic and continued supply scarcity in numerous regions, many studies have sought to identify alternative strategies to minimize risk of developing severe COVID-19 following SARS-CoV-2 infection and also to treat severe COVID-19. In addition to evaluations of existing pharmacologic agents (*e.g.*, ivermectin, hydroxychloroquine, azithromycin, and dexamethasone), vitamin and nutrient supplementation has been widely studied. Global mortality rate differences associated with latitude and clinical observations of low serum 25-hydroxyvitamin D levels among hospitalized COVID-19 patients has perhaps garnered greatest attention^22^, but we did not observe a significant association between genetic determinants of vitamin D levels and COVID-19 severity. However, we observed nominally significant protective effects for less-studied nutrient-related traits, including magnesium, calcium, retinol, and vitamin E. A combined vitamin D/magnesium/vitamin B12 combination was associated with a reduction in the proportion of elderly COVID-19 patients requiring oxygen support and intensive care support in a small prospective cohort^23^, and lower plasma retinol levels have also been observed in hospitalized COVID-19 patients^24^. We did not observe a significant association between serum Vitamin D levels and risk for COVID-19 or severe outcomes. Vitamin E levels have not been widely examined in the context of COVID-19, but deficiency is frequently associated with intestinal malabsorption rather than dietary insufficiency and thus may reinforce the observed genetic correlation between COVID-19 disease severity and diverticular disease in our analyses. In our study, we do observe an association between higher levels. Further, we observe protective associations for both HDL and serum concentration of apolipoprotein A, a major component of the HDL complex involved in clearing fat. HDL is involved in vitamin E absorption and contains approximately 40% of circulating α-tocopherol, the main dietary source of vitamin E^25^.

Integration and harmonization of extant large-scale GWAS datasets has become a popular approach to reveal novel epidemiologic associations. Still, access to individual-level GWAS datasets remains limited, because of data use restrictions. The LDSR method does not require individual-level genotype data or LD pruning and can quantify the shared genetic architecture of traits having undergone GWAS analysis. However, LDSR analysis assumes absence of population stratification in the underlying summary statistics used and necessitates incorporation of GWAS data from populations expected to have similar genomic architecture. This assumption restricted our analysis to use of GWAS data from British-ancestry individuals, limiting our ability to make conclusions about the shared genetic architectures among other racial/ethnic groups. Given that COVID-19 disease severity has been associated with racial/ethnic background, as well as socioeconomic status and somatic well-being, it is imperative that efforts be made to enrich future genetic epidemiology studies for participants of non-European descent to expand generalizability of results. Interpretation of our results is also limited by the strong correlation between many of the traits studied with BMI and smoking behaviors. Although we made efforts to limit the impact of BMI and smoking-associated genetic variation by excluding known loci from LDSR analysis, such sensitivity analyses cannot account for polygenic contributions not yet having reached genome-wide statistical significance in prior research.

To estimate cross-trait genetic correlation, we restricted the range of UKBB traits with an arbitrary threshold of h^2^ ≥ 1% to improve reliability. For instance, there were additional subtypes of diabetes derived from the various medical records in UKBB and their estimates of SNP-heritability showed h^2^_Type 1 Diabetes_ = 0.3% and h^2^_Type 2 Diabetes_ = 0.4%. Therefore, we did not include results from them. The type of diabetes reported in Table 1, described as “Diabetes diagnosed by doctor” (UKBB Field Identifier:2443), is not specified for the type of diabetes, but given the age of participants and the general prevalence of T2D versus T1D, the association between diabetes and COVID-19 severity is ostensibly driven by the shared genetic architecture between COVID-19 severity and the genetic architecture of T2D. Furthermore, LDSR analysis relies on the common genetic variants with MAF > 1% and therefore it can fail to capture the SNP-heritability on the observed scale due to underlying low-frequency or rare variants^4^. If a polygenic trait in UKBB shows a significant genetic correlation with COVID-19 severity, this does not imply a causal association. Both the tested trait and COVID-19 severity risk may be jointly influenced by an unmodeled trait that is independently associated with each^19^. Although our study relies on associations with common genetic variants (generally, MAF > 1%), the inclusion of additional rare genetic variants might be valuable as it could increase the overall trait heritability being modeled. Inferences from LDSR rely on normality assumptions that may be violated when rare variants are studied, and we therefore restricted analysis to more common variants. Additionally, LDSR does not explicitly model confounding effects which can arise when studying multiple correlated traits. Therefore, the method identifies novel associations that can be further studied using Mendelian Randomization or direct analyses of the nominated trait phenotypes for further confirmation of causal relationships. LDSR is a useful approach for identifying potential novel associations that will warrant further epidemiological analysis to tease apart causal associations from associations that are influenced by confounding.

Our findings support previously identified risk factors for severe COVID-19 illness, including elevated BMI, diabetes, and numerous pulmonary conditions (*e.g.*, COPD, reduced FEV, shortness of breath during mild activity). We also observe protective associations between the genetic underpinnings of COVID-19 severity and that of non-smoking, serum albumin, apolipoprotein A, HDL cholesterol level, and several nutrients. Further studies using Mendelian randomization approaches may help to dissect causal associations between COVID-19 disease severity and these traits, potentially nominating targets for therapeutic intervention.

## Supplementary Information

Supplementary Information.

## Data Availability

The datasets supporting the conclusions of this article are publicly available in the COVID-19 HGI website for COVID-19 severity GWAS summary statistics [https://www.covid19hg.org/results/r5/] and Neale’s lab repository for UK Biobank GWAS summary statistics [https://github.com/Nealelab/UK_Biobank_GWAS], and therefore no approvals were required.
